# Chemical Characterization and Bioactivity of Extracts from *Thymus* *mastichina*: A *Thymus* with a Distinct Salvianolic Acid Composition

**DOI:** 10.3390/antiox9010034

**Published:** 2019-12-31

**Authors:** Meriem Taghouti, Carlos Martins-Gomes, Judith Schäfer, João A. Santos, Mirko Bunzel, Fernando M. Nunes, Amélia M Silva

**Affiliations:** 1 Centre for Research and Technology of Agro-Environmental and Biological Sciences (CITAB), University of Trás-os-Montes and Alto Douro (UTAD), 5001-801 Vila Real, Portugal; myriam@utad.pt (M.T.); camgomes@utad.pt (C.M.-G.); jsantos@utad.pt (J.A.S.); 2 Food and Wine Chemistry Laboratory, Chemistry Research Centre—Vila Real (CQ-VR), University of Trás-os-Montes and Alto Douro (UTAD), 5001-801 Vila Real, Portugal; 3 Department of Food Chemistry and Phytochemistry, Institute of Applied Biosciences, Karlsruhe Institute of Technology (KIT), Adenauerring 20a, Building 50.41, 76131 Karlsruhe, Germany; judith.schaefer@kit.edu (J.S.); mirko.bunzel@kit.edu (M.B.); 4 Department of Physics, School of Sciences and Technology, UTAD, 5001-801 Vila Real, Portugal; 5 Department of Chemistry, School of Life Sciences and Environment, UTAD, 5001-801 Vila Real, Portugal; 6 Department of Biology and Environment, School of Life Sciences and Environment, University of Trás-os-Montes and Alto Douro (UTAD), 5001-801 Vila Real, Portugal

**Keywords:** *Thymus mastichina*, phenolic profiling, aqueous decoction, hydroethanolic extract, salvianolic acid A isomer, salvianolic acid isomer B/E isomer, anti-proliferative activity, radical scavenging activity, antioxidant

## Abstract

*Thymus mastichina*, also called mastic thyme or Spanish marjoram, is endemic to the Iberian Peninsula, where it is widely used in folk medicine especially for treating digestive and respiratory systems disorders, and as a condiment to season olives. This work describes for the first time the detailed phenolic composition of exhaustive hydroethanolic extracts and aqueous decoctions of *Thymus mastichina*. Unlike other species of the *Thymus* genera, *Thymus mastichina* extracts contain high amounts of salvianolic acid derivatives, with salvianolic acid A isomer being the main derivative. This isomer was identified in extracts from *Thymus mastichina* for the first time. Also, an undescribed salvianolic acid derivative in *Thymus mastichina* was identified and its structure was tentatively described. Extracts from *Thymus mastichina* showed significant scavenging activity of 2,2-azino-bis (3-ethylbenzothiazoline-6-sulfonic acid) diammonium salt (ABTS) radical cation, hydroxyl, and nitric oxide radicals. The anti-proliferative effect of both *T. mastichina* extracts were tested against Caco-2 and HepG2 cells; the hydroethanolic extract showed a high anti-proliferative activity against Caco-2 cells compared to HepG2 cells (at 24 h exposure, the concentration that inhibits 50% of proliferation, IC_50_, was 71.18 ± 1.05 µg/mL and 264.60 ± 11.78 µg/mL for Caco-2 and HepG2, respectively). Thus, these results make this species a promising candidate for further investigation of its anti-tumoral potential. Therefore, *Thymus mastichina* can be potentially used as a functional food (used as a decoction or herbal tea) or as a source of bioactive ingredients with antioxidant and anti-proliferative properties.

## 1. Introduction

The increasing interest in phenolic compounds as natural ingredients for food additives and as health promoters resulted in a deeper investigation of many plant species that are widely used in folk medicine (e.g., [1,2,3]). Ethnobotanical surveys, particularly from the Mediterranean area, often describe the medicinal uses of plants from the genus *Thymus*, a member of the Lamiaceae family, due to their high anti-microbial [4], anti-oxidant [5,6], anti-inflammatory [7,8,9], and anti-proliferative [6,10,11,12] activities. Some of the observed bioactivities have already been correlated with the chemical composition of the different *Thymus* species, with an emphasis on various terpenoids and phenolic compounds [9,11,13,14].

*Thymus mastichina* (*T. mastichina*), also called mastic thyme, Spanish marjoram, or white thyme, is endemic to the Iberian Peninsula where it is traditionally used for treating digestive, respiratory, and rheumatic disorders [15,16,17]. Also, it is used as a condiment (e.g., to season olives and to aromatize olive oil) and as a herbal infusion in the food industry, and as a source of essential oil in the cosmetic and perfume industries [17,18]. Methanolic extracts of various *T. mastichina* samples collected in Spain contained rosmarinic acid as the main phenolic compound and exhibited good antioxidant activities [17,19]. The observed bioactivities may be attributed to the chemical composition of *T. mastichina*. Isolated phenolic compounds of dichloromethane and ethanolic *T. mastichina* extracts were tested against HCT 116 (human colorectal carcinoma) cell line, evidencing anti-tumoral activity, with ursolic acid being the most active component [14]. In the literature, the main polyphenolic compounds identified in *T. mastichina* extracts are rosmarinic acid, methoxysalicylic acid, apigenin, kaempferol, luteolin, chlorogenic acid, caffeic acid, and derivatives of luteolin and apigenin [14,19,20]. In addition, terpenoids, such as oleanolic and ursolic acids [14], were identified. Compared to the *T. mastichina* essential oil’s composition, which is well characterized, the phenolic profile is poorly investigated and described, as to date, there is only one study dedicated to the phenolic composition of *T. mastichina*, and most of the peaks present in the chromatogram remained unidentified [17].

Further application of *T. mastichina* as a functional food and/or as a source of bioactive ingredients require an extensive characterization of the phenolic composition to correlate them with potentially claimed bioactivities. The importance of functional foods, nutraceuticals, and other natural health products has been well recognized in connection with health promotion and disease risk reduction [21]. A nutraceutical is “a food or part of a food that provides benefits health in addition to its nutritional content” that can be effectively used by inclusion in the daily diet as they combine both nutritional and beneficial health properties of natural bioactive compounds. One of the main differences between nutraceuticals and pharmaceuticals is that pharmaceuticals are usually made of one single substance and nutraceuticals are made of a pool of substances [22]. Active substances extracted from plants (phytocomplexes) or of animal origin, when extracted, concentrated, and administered in a suitable pharmaceutical form, can create a very promising tool to prevent and/or support the therapy of some pathologic conditions given their proven clinical efficacy [23]. Thus, this study aimed to characterize the chemical composition of two *T. mastichina* extracts, one obtained by aqueous decoction and the other by exhaustive hydroethanolic extraction. Furthermore, their bioactivity by in vitro antioxidant methods as well as their anti-proliferative activity using Caco-2 (human colon adenocarcinoma cell line) and HepG2 (human hepatocellular carcinoma cell line) cells was evaluated. These cell lines were chosen because *T. mastichina* is used as condiment (aerial parts of the plant) and also as an infusion herb. Therefore, these two cell lines mimic (as an in vitro approach) the effect of plant components’ interaction with intestinal tract tissues, the colon, during absorption and with hepatic tissues, as a result of the first passage of absorbed components.

## 2. Materials and Methods

### 2.1. Standards and Reagents

Dulbecco’s modified eagle medium (DMEM), Trypsin-EDTA (EDTA—ethylenediaminetetraacetic acid), sodium pyruvate penicillin, streptomycin, l-glutamine, fetal bovine serum (FBS), and versene were obtained from Gibco (Alfagene, Invitrogen, Portugal). Alamar Blue^®^ was purchased from Invitrogen, Life-Technologies (Porto, Portugal). Formic acid, acetic acid, ethanol, and methanol were HPLC (High performance liquid chromatography) or MS (Mass Spectrometry) grade according to the analysis and were purchased from Sigma-Aldrich/Merck (Algés, Portugal). Commercial standards of rosmarinic acid, catechin, salvianolic acid A, salvianolic acid B, ursolic acid, and luteolin were obtained from Sigma-Aldrich/Merck (Algés, Portugal). Oleanolic acid was obtained from Santa Cruz Biotechnology Inc. Caffeic acid and quercetin-3-*O*-glucoside were obtained from Extrasynthese^®^ (Genay, France). Sodium nitroprusside, sulfanilamide, *N*-(1-naphthyl)ethylenediamine dihydrochloride, 2,2-azino-bis (3-ethylbenzothiazoline-6-sulfonic acid) diammonium salt (ABTS), (±)-6-hydroxy-2,5,7,8-tetramethylchromane-2-carboxylic acid (Trolox), potassium persulfate, Folin-Ciocalteu’s reagent, ethylenediaminetetraacetic acid (EDTA), sodium nitrite, ascorbic acid sodium molybdate, aluminum chloride (III), thiobarbituric acid (TBA), trichloroacetic acid (TCA), 2-deoxy-d-ribose, and hydrogen peroxide 30% solution were purchased from Sigma-Aldrich/Merck (Algés, Portugal). Other salts and reagents not mentioned were obtained from Sigma-Aldrich/Merck (Algés, Portugal).

### 2.2. Plant Material

Fresh plants were kindly supplied by ERVITAL^®^ (Plantas Aromáticas e Medicinais, Lda; Mezio, Portugal). Aerial parts of *T. mastichina*, grown in organic farming conditions were collected in October 2014. Part of the plant material served for authentication by the botanical garden office at the University of Trás-os-Montes and Alto Douro (UTAD, Vila Real, Portugal), and a voucher specimen (Voucher N. HVR21091) was deposited. Collected plants were rinsed with distilled water, weighted, and frozen (−20 °C) upon arrival. After the lyophilization process (Dura Dry TM μP freeze-drier; −45 °C and 250 mTorr), the samples were properly stored until further extraction and analysis.

### 2.3. Preparation of Extracts

Lyophilized aerial parts of *T. mastichina* were ground in a blender and extracted using two extraction methods: Aqueous decoction (AD) and hydroethanolic (HE) extraction, as detailed in [9]. Briefly, 0.5 g of freeze-dried ground plant material were used for each extraction. The decoction was performed by adding distilled water (150 mL) to the plant material, and the suspension was heated up to 100 °C under agitation and boiled for 20 min. After cooling to room temperature, the mixture was filtered, concentrated to 100 mL (rotary evaporator, 35 °C), frozen, and freeze-dried. HE exhaustive extraction was performed as three-step sequential extraction at room temperature by using 50 mL of ethanol:water (80:20, *v*/*v*) that were added to the plant material. The mixture was agitated (orbital shaker, 150 rpm, one hour) and then centrifuged. The supernatant was filtered and collected, then 50 mL of ethanol:water (80:20, *v*/*v*) solution was added to the pellet. After repeating the extraction three times, the three supernatants were combined. In both extraction methods, the extracts were filtered twice (Whatman n° 4 filter and fiberglass filter (1.2 μm; acquired from VWR International Ltd., Alfragide, Portugal)). Both extracts were concentrated in a rotary evaporator (35 °C). The extracts were then lyophilized and weighed to calculate the yields.

### 2.4. Total Phenolic Compound, Total Flavonoid, and Ortho-Diphenol Contents

Total phenolic content (TPC) was performed according to the Folin–Ciocalteau assay, following the method described by Machado et al. (2013) [24]. *T. mastichina* extract (0.1 mg/mL; 1 mL) was mixed with 0.5 mL of Folin-Ciocalteau reagent, 1 mL of Na_2_CO_3_ (7.5%), and 7.5 mL of distilled water. The mixture was incubated at room temperature for 1 h, and absorbance was read at 725 nm using a spectrophotometer (PerkinElmer, Lambda 25 UV/VIS Spectrometer; Waltham, MA, USA). Caffeic acid was used as the standard and TPC was expressed as caffeic acid equivalents (mg CA eq./g freeze-dried plant or mg CA eq./g extract).

Total flavonoid contents (TFC) were determined using the aluminum chloride colorimetric method [20]. Extract solution (1 mL; 0.5 mg/mL) was incubated for 5 min with 150 μL of NaNO_2_ (5%), at room temperature, followed by the addition of 150 μL AlCl_3_ (10%). After 6 min of incubation, 1 mL of NaOH (1 M) was added and the absorbance was read at 510 nm. Catechin was used as the standard and TFC was expressed as mg catechin equivalents (mg C eq./g dry plant or mg C eq./g extract).

*Ortho*-diphenol (ODP) contents were determined by using the sodium molybdate colorimetric method described by Machado et al. [24]. Extracts (4 mL; 0.1 mg/mL) were mixed with 1 mL of sodium molybdate (5%). The mixture was incubated (15 min at room temperature), and absorbance was measured at 370 nm. Caffeic acid was used as the standard and the ODP content was expressed as mg caffeic acid equivalents (mg CA eq./g dry plant or mg CA eq./g of extract).

### 2.5. Profiling and Quantification of Individual Phenolic Compounds by High Performance Liquid Chromatography with Diode Array Detector (HPLC-DAD) and High Performance Liquid Chromatography with Electrospray Ionization and Tandem Mass Spectrometry Detection (HPLC-ESI-MS^n^)

Phenolic compounds were identified by RP-HPLC-DAD and RP-HPLC-ESI-MS^n^. RP-HPLC-DAD analysis was carried out using an Ultimate 3000 HPLC (Dionex, Sunnyvale, CA, USA) equipped with an Ultimate 3000 pump, a WPS-3000 TSL Analyt auto-sampler, and an Ultimate 3000 column compartment coupled to a PDA-100 photodiode array detector. A C18 column (ACE 5 C18; 250 mm × 4.6 mm; particle size 5 μm) was used for the chromatographic separation. The temperature was held at 35 °C during the run and UV-Vis detection was performed between 200 and 600 nm. HPLC conditions were used as previously described [12]. Chromeleon software (Version 7.1; Dionex, Sunnyvale, CA, USA) was used for data acquisition, peak integration, and analysis.

LC-MS^n^ analysis was carried out using a Thermo Scientific system equipped with a Finnigan Surveyor Plus auto-sampler, photodiode array detector and pump, and an LXQ Linear ion trap detector. The column used was a Luna C18 (2) (250 mm × 4.6 mm, 5μm; Phenomenex (Aschaffenburg, Germany)) kept at 40 °C. Program conditions, eluents, flow rate, and injection volume were used as described by Taghouti et al. [12]. Electrospray ionization (ESI) was performed in negative mode (capillary temperature: 350 °C; spray voltage: −4 kV; capillary voltage: −5 kV). Mass detection was performed in the range 100–1000 *m*/*z*.

Peak identification was based on UV-VIS spectra, retention time, and mass spectra compared to commercial standards and/or literature data. For quantification of phenolic compounds, calibration curves of available standards were prepared [12]. When commercial standards were not available, the quantification performed by using the aglycones or standard compounds with structural similarity. Quercetin-(?)-*O*-hexoside was quantified as quercetin-3-*O*-hexoside; luteolin-(?)-*O*-hexoside and chrysoeriol-(?)-*O*-hexoside were quantified as luteolin; salvianolic acid A isomer was quantified as salvianolic acid A; salvianolic acid B/E isomer 2 was quantified as salvianolic acid B; and salvianolic acid K and I were quantified as rosmarinic acid.

### 2.6. Determination of Oleanolic Acid and Ursolic Acid in Hydroethanolic Extracts

Identification and quantification of ursolic acid (UA) and oleanolic acid (OA) were performed in HE extracts using RP-HPLC according to the method described previously by Taghouti et al. [12].

### 2.7. In Vitro Antioxidant Activity Assessment

#### 2.7.1. ABTS Radical Cation Decolorization Assay

ABTS•^+^ scavenging assay was performed as described by Machado et al. [24]. A mixture of equal volumes of ABTS with potassium persulfate (K_2_S_2_O_8_) was allowed to react for 15 to 16 h in the dark at room temperature to produce the ABTS•^+^ radical. The radical solution was diluted with acetate buffer (20 mM, pH 4.5) to obtain an absorbance of 0.700 ± 0.02 (at 734 nm). To determine the scavenging activity of *T. mastichina* extracts, 200 μL of extracts (0.1 mg/mL) were added to 2 mL of ABTS•^+^ solution, and absorbance was read after 15 min at 734 nm. Trolox ((±)-6-hydroxy-2,5,7,8-tetramethylchromane-2-carboxylic acid) was used as a standard antioxidant and the scavenging potential was expressed as Trolox equivalents (mmol Trolox/g dry plant or mmol Trolox/g extract).

#### 2.7.2. Hydroxyl Radicals Scavenging Assay

Hydroxyl radical (OH•) scavenging activity was performed as described by Taghouti et al. [12]. To 0.5 mL of extract solution (0.1 mg/mL), equal volumes (100 μL) of deoxyribose (20 mM), FeCl_2_ (1 mM), ascorbic acid (1 mM), H_2_O_2_ (10 mM), and 400 μL of phosphate buffer solution (20 mM; pH 7.4) were added. A second equal sample set was prepared to contain an additional 100 μL of EDTA (1 mM). Both experimental sets were incubated 1 h at 37 °C. Then, 1.5 mL of TBA (thiobarbituric acid 5% prepared in TCA (trichloroacetic acid, 10%)) was added, followed by 15 min of incubation, at 100 °C. The absorbance was read at 532 nm and a blank was used as the control (same mixture as described above, with 0.5 mL of H_2_O replacing the extract). The OH• scavenging activity was expressed as percentage inhibition using Equation (1):

(1) $$Inhibition\;\left(\%\right)=\frac{\mathrm{Blank}\;\mathrm{abs}-\mathrm{Sample}\;\mathrm{abs}\;}{\mathrm{Blank}\;\mathrm{abs}\;}\times 100$$

#### 2.7.3. Nitric Oxide Radical Scavenging Assay

Nitric oxide radical (NO•) scavenging activity was performed as described by Sreejayan and Rao [25]. Briefly, a solution of sodium nitroprusside (5 mM) was prepared in a phosphate buffer (0.1 M H_3_PO_4_; pH 7.4) and was oxygenated by purging with air for 15 min. Extract solution (0.5 mL; 1 mg/mL) was added to 4.5 mL of sodium nitroprusside solution and incubated at 35 °C for 2 h. NO• quantification was performed using Griess reagent (equal volumes of 1% sulfanilamide (in 5% H_3_PO_4_) and 0.1% n-alpha-naphthyl-ethylenediamine (in water)). To 1 mL of samples (sodium nitroprusside solution + extract), 1 mL of Griess reagent was added. After 3 min of incubation, absorbance was measured at 546 nm. Sodium nitrite was used as the positive control. NO• scavenging activity was expressed as the inhibition percentage; for the control (blank), H_2_O was used to replace the extract. The inhibition percentage was calculated according to Equation (1).

### 2.8. In Vitro Cell Viability Assay

Caco-2 (human colon adenocarcinoma cell line; Cell Lines Service, Eppelheim, Germany) and HepG2 (human hepatocellular carcinoma cell line; ATCC^®^ Number: HB-8065^TM^, a gift from Prof. C. Palmeira CNC-UC, Portugal) cells were cultured in Dulbecco’s modified eagle media (DMEM) supplemented with 10% (v/v) fetal bovine serum (FBS), 1 mM L-glutamine, and antibiotics (100 U/mL of penicillin and 100 μg/mL streptomycin). Cells were maintained, in a Binder incubator, at 37 °C in 5% CO_2_/95% air conditions with controlled humidity, and handled as by Severino et al. [26].

The anti-proliferative effect of extracts was performed with the Alamar Blue (resazurin) assay^®^ (Alfagene, Lisbon, Portugal) [27]. Resazurin is a non-toxic, water-soluble, cell-permeable redox indicator that can be used to monitor viable cell numbers. Cellular metabolic activity results in dye conversion from the oxidized form (resazurin; blue) into the reduced form (resorufin; pink), with the process being accompanied by a color change. The percentage of reduced Alamar Blue can be considered proportional to the cell viability. Briefly, cells were seeded in 96-well plates at a density of 5 × 10^4^ cells/mL (100 μL/well) in culture media. After 48 h of culture for cell adhesion, the culture media was replaced with 100 μL of test solutions, prepared by dilution of respective extracts, at 10 mg/mL (stock solutions) in FBS-free DMEM to achieve the desired test solution concentrations (range 50–500 μg/mL). Cells were exposed to extracts during 24 or 48 h. After exposure, extract solutions were removed and 100 μL of Alamar Blue solution (10% (*v*/*v*), in FBS-free culture media) were added to each well. After 5 h of incubation, the absorbance was read at 570 (reduced form) and 620 nm (oxidized form) using a Multiskan EX microplate reader (MTX Labsystems, Bradenton, FL, USA). Results were expressed as cell viability (% of control; non-treated cells), calculated as described by Andreani et al. [27]. Control cells were submitted to all procedures but only received FBS-free DMEM, instead of the extract, and were exposed to Alamar Blue solution simultaneously with other cells.

### 2.9. Statistical Analysis

Three extractions were carried out for each extraction method of extraction and analyses were performed in triplicate. The concentration that inhibits 50% of cell viability, i.e., the IC_50_ values were calculated as described by Silva et al. [28]. Analyses of variance (ANOVA) followed by Tukey’s multiple test (α = 0.05) were performed to analyze both differences in chemical composition and effects on cell viability. Correlations were evaluated using Pearson’s coefficient (significant if *p* < 0.05). GraphPad Prism version 7 (GraphPad Software Inc, CA, USA), Microsoft Office Excel 365 (Microsoft Corporation, WA, USA), and Statistica 12.0 (Dell Software, TE, USA) were used for graph construction and statistical analysis.

## 3. Results and Discussion

### 3.1. Extract Yield and Chemical Composition of Extracts

In this study, two methods of extraction were selected to obtain *T. mastichina* extracts: The aqueous decoction (AD) aiming to mimic the common procedure of beverage preparation for human consumption, as this plant is also used as herbal tea, and the exhaustive hydroethanolic (HE) extraction was chosen as a method to obtain all possible “free” phenolic compounds. In a previous work, performing successive hydroethanolic extractions showed that 99% of the total extractable compounds were extracted in the first three extractions [9]. As expected, the exhaustive HE procedure resulted in higher yields than the one-step AD procedure (~14% vs. ~9% (*w*/*w*), for HE and AD, respectively, Table 1), nevertheless, the AD method was able to extract approximately 68% of the material extracted with exhaustive HE extraction. These results are in line with those described by Taghouti et al. [12] for *T. pulegioides* and slightly lower than those obtained by Martins-Gomes et al. [9] for *T. carnosus*. The exhaustive HE extraction also resulted in higher extraction yields of total phenolic compounds (~1.96 times higher than the yield obtained by AD extraction, Table 1). The efficiency of the three-step HE extraction in yielding higher TPC contents was observed for *T. pulegioides* [12] and *T. carnosus* [9], too. Using different extraction conditions (50% methanol; 1 h ultra-sound bath, at room temperature), Méndez-Tovar et al. [17] obtained a wide range of TPC contents in *T. mastichina* (from ~7 to ~56 mg CA eq./g dry plant) during the characterization of 14 different wild plant populations across Spain. The results found here (Table 1) for this species are within these values, although the extraction and geographical origins are different. Aqueous extraction of *T. mastichina* grown in northern Portugal yielded lower TPC contents (47 to 60 mg CA eq./g extract), probably due to saturation of the extraction solution as 1 g of dry material was used per 50 mL of water. However, the extraction procedure using other solvent mixtures (ethanol/methanol) resulted in similar TPC values (109 to 165 mg CA eq./g extract) [16] than the ones observed here (Table 1). Methanol extracts of *T. mastichina* obtained by applying the soxhlet method [19] and by repeated maceration periods [29] contained lower TPC contents than our extracts (Table 1). The same trend was observed for the TFC and ODP contents extracted with exhaustive HE and AD. Although the relative portions of TPC, TFC, and ODP in the extracts obtained by HE and AD extraction from *T. mastichina* were similar to those found in extracts from *T. pulegioides* [12] and *T. carnosus* [9], the absolute amount was lower, especially when compared to *T. pulegioides*, where an almost double amount was extracted for both the AD and HE extraction methods.

### 3.2. Phenolic Compound Profiles in Aqueous Decoction and Hydroethanolic Extracts

**Figure 1:**
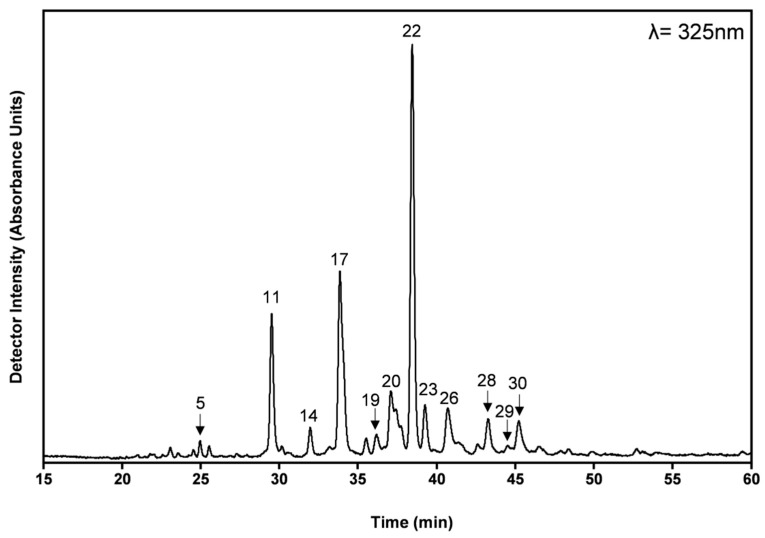
Phenolic profile of *Thymus mastichina*. HPLC-DAD chromatogram of hydroethanolic extract. For peak identification, please refer to Table 2.

The phenolic profile and the individual phenolic compounds present in the HE extract, as well as their concentrations, are shown in Figure 1 and Table 2. Table 2 also contains the phenolic compounds present in AD extracts and respective quantification. The relative portion of each phenolic compound determined by HPLC-DAD is in accordance with the results obtained from the colorimetric methods discussed above (Table 1). To the best of our knowledge, this is the first report on the detailed phenolic composition of *T. mastichina.* The phenolic composition of *T. mastichina* extracts is comparable to the phenolic composition previously reported in the literature for the *Thymus* genera [30], with rosmarinic acid being the main phenolic compound present in both HE and AD extracts (Table 2). Rosmarinic acid represents 34% to 40% of the total phenolic compounds extracted by the HE and AD methods, respectively. Compared to other *Thymus* species, using the same extraction methods, these portions are higher than those obtained for *T. carnosus* (rosmarinic acid = 6% and 20%, AD and HE, respectively [9]) and identical to those obtained for *T. pulegioides* (rosmarinic acid = 35% and 47%, AD and HE, respectively [12]). Although, when slightly different extraction methods were used, the rosmarinic acid content (Table 2) is comparable to other *T. mastichina* extracts [17]. Aqueous decoction from *T. zygis*, *T. pulegioides*, and *T. fragrantissimus* were composed by 52%, 36%, and 64% of rosmarinic acid, respectively [31], and high contents in rosmarinic acid were also reported in *Thymus algeriensis* [32], indicating that rosmarinic acid is a relevant phenolic compound in most *Thymus* species.

**Figure 2:**
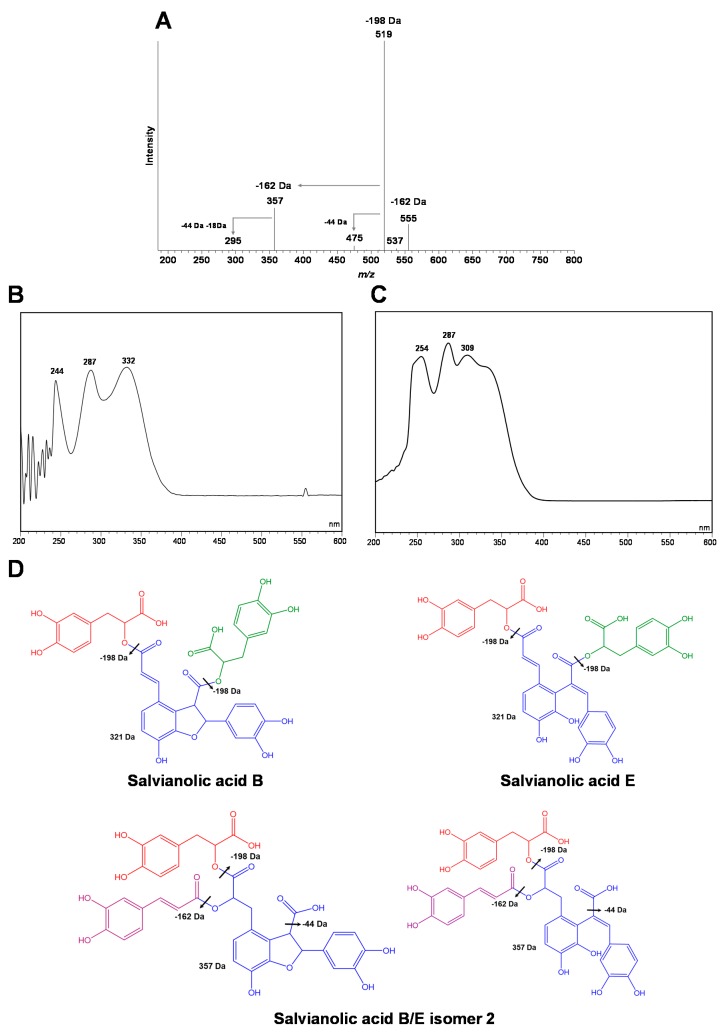
ESI-MS^2^ (*m*/*z* = 717) (**A**) and UV-VIS (**B**) spectra of salvianolic acid B/E isomer 2, UV-VIS spectrum of salvianolic acid B standard (**C**) and fragmentation of salvianolic acids B and E with two possible structures, and respective fragmentation, of salvianolic acid B/E isomer 2 (**D**).

*T. mastichina* extracts are composed of relatively high amounts of salvianolic acid derivatives, including salvianolic acid K and I (only detected and not quantified in AD extract), and two isomers of salvianolic acid A and B/E isomer 2. These salvianolic acid derivatives together account for 38% and 32% of the total phenolic acids extracted by HE and AD extractions, respectively (Table 2). Also, a salvianolic acid F derivative and a salvianolic acid K isomer were detected but not quantified due to small amounts. The presence of salvianolic acid derivatives in other *Thymus* species has been previously described in the literature [9,12,31,32,33,34,35]. Except for *T. carnosus* [9], and *Thymus algeriensis* [32], their portion is normally represented either as <5% of the total phenolic compounds extracted [12,31] or they could not be identified [13,17]. Salvianolic acid A isomer was identified as the main salvianolic acid derivative in the extracts from *T. mastichina*. This isomer was first described in the *Thymus carnosus* species [9]. In addition, another salvianolic acid isomer was detected in the extracts of *T. mastichina* that eluted before salvianolic acid A isomer (Figure 1). The mass/charge (*m*/*z*) ratio of the pseudo-molecular ion (*m*/*z* = 717) is identical to salvianolic acid B and E, and the isomer was therefore named salvianolic acid B/E isomer 2. One isomer of salvianolic acid B/E has been previously described in *Salvia miltiorrhiza* [36]. Additionally, salvianolic acid K, B (isomer 1 and 2), and I (isomer 1) have been described in decoctions of *Salvia apiana* and *Salvia farinacea* var. Victoria Blue [37]. However, its structure was not deduced and confirmed by further analysis. This isomer showed a retention time higher than that of salvianolic acid B [36]; however, in our case, the retention time of the new isomer was lower than that of salvianolic acid B (Figure 1), which in our chromatographic system presented a retention time of 38.667 min (not shown). The salvianolic acid B/E isomer 2 represents 9.5% and 8.8% of the total phenolic compound extracted by exhaustive HE and AD. As can be observed in Figure 2, the ESI-MS/MS spectra of the salvianolic acid B/E isomer 2 identified in *T. mastichina* is clearly different from that of salvianolic acid B and E [36,38,39]. The fragmentation scheme of salvianolic B and E and the main ions present in MS/MS spectra, according to the literature, are shown in Figure 2D. Also, there is a difference between the UV-Vis spectra of the salvianolic acid B/E isomer 2 and those of salvianolic acid B standard (Figure 2C) and salvianolic acid E [38]. There is a clear difference between the fragmentation patterns of salvianolic acid B/E isomer 2 and those of salvianolic acid B and E. The fragment ion with *m*/*z* = 555 observed in the MS spectrum of salvianolic acid isomer 2, corresponding to the loss of 162 Da from the molecular ion *m*/*z* = 717, may be attributed to the loss of a caffeic acid residue. The loss of 162 Da is absent in the MS/MS fragmentation of salvianolic acid B and E [36,39]. The main fragment ion of salvianolic acid B/E isomer 2 is observed at *m*/*z* = 519, which can be attributed to the loss of salvianic acid (loss of 198 Da from the molecular ion with *m*/*z* = 717). This fragment ion is also observed in the MS/MS spectra of salvianolic acids B and E, too [36,39]. The presence of caffeic acid residue in the salvianolic acid B/E isomer 2 is also supported by the presence of the fragment ion with *m*/*z* = 357, resulting from the loss of 162 Da from *m*/*z* = 519. This fragment ion with *m*/*z* = 357 is only present in the MS/MS spectra of this new compound. In the MS/MS spectra of salvianolic acids B and E, the fragment ion with *m*/*z* = 321 is observed due to the loss of 198 Da from the fragment ion at *m*/*z* = 519. Fragment ions with *m*/*z* = 475 and *m*/*z* = 295, corresponding to the loss of 44 and 62 Da from ions with *m*/*z* = 519 and *m*/*z* = 357, are only present in the MS/MS spectra of salvianolic acid B/E isomer 2 when compared to the MS/MS spectra of salvianolic acids B and E. The loss of 44 Da can be attributed to the loss of HCOOH, indicating a free carboxylic acid structure in the molecule. The loss of 62 Da may be due to the loss of HCOOH + H_2_O. The additional loss of H_2_O (18 Da) supports the presence of a hydroxyl group in the salvianolic acid B/E isomer 2 after the elimination of salvianic acid and caffeic acid residues. Also, there is a significant difference between the UV-Vis spectra of the salvianolic acid B/E isomer 2 and that of the salvianolic acid B standard (Figure 2C) and salvianolic acid E [38], with the disappearance of the peak located at 309 nm being present as the peaks at 287 and 332 nm also present in salvianolic acids B and E, the last one as a shoulder due to peak overlapping. Taking into account this fragmentation pattern observed for the salvianolic acid B/E isomer 2 present in *T. mastichina*, and the known fragmentation pattern of other salvianolic acid derivatives already known, the tentative structure of the salvianolic acid B/E isomer 2 is presented in Figure 2D.

Besides rosmarinic acid and salvianolic acid derivatives, the hexoside derivatives of quercetin, luteolin, and chrysoeriol were quantified in the extracts of *T. mastichina* (Table 2). These flavonoids were previously described for other *Thymus* species, too. The quantified flavonoids represent 28% and 26% of the total phenolic compounds extracted by HE and AD, respectively. Luteolin hexoside, which is probably a luteolin glucoside due to a previous report of this compound in *T. mastichina* populations from Spain [17], accounted for almost 50% of the quantified flavonoids in our HE extracts of *T. mastichina.* Also, other flavonoids were identified but not quantified, namely derivatives of eriodictyol, quercetin, and luteolin (Table 2).

These results show that the phenolic profiles of the extracts from *T. mastichina* (Table 2) are comparable to the phenolic profiles described in the literature for various *Thymus* species (e.g., [9,12,13,40]). However, extracts from *T. mastichina* contain lower amounts of extractable phenolic compounds compared to other *Thymus* species. In addition, two salvianolic acid isomers were quantitated in significant amounts. The first isomer, salvianolic acid A isomer, was previously described for *T. carnosus*, and the second isomer, salvianolic acid B/E isomer 2, is described here for the first time in *Thymus* species.

### 3.3. Oleanolic Acid and Ursolic acid Contents

Oleanolic (OA) and ursolic (UA) acids are two triterpenes that are commonly described as being present in the alcoholic extracts from various *Thymus* species, for example, from *T. serpyllum* (3.7 and 13.9 mg/g dry plant, for OA and UA, respectively [41]) and *T. carnosus* (9.9 and 18.7 mg/g dry plant, for OA and UA respectively [9]). However, these triterpenes were not detected in the HE extracts from *T. mastichina.*

### 3.4. In Vitro Antioxidant Activity

Extracts of *T. mastichina* showed a significant scavenging activity of ABTS radical cation. As expected, significantly higher scavenging activity values were obtained for the HE extracts (~1.48 mmol Trolox eq./g extract; Table 1) compared to the AD extracts (~0.96 mmol Trolox eq./g extract, Table 1). The values expressed as Trolox eq./g dry plant (Table 1) were lower than those found by Taghouti, Martins-Gomes, Schafer, Felix, Santos, Bunzel, Nunes, and Silva [12] for HE and AD extracts of *T. pulegioides* (0.15 and 0.34 mmol Trolox eq./g dry plant, respectively). However, they are in the same range as those observed for the extracts of *T. carnosus* (0.14 and 0.21 mmol Trolox eq./g dry plant, respectively) [9].

For the hydroxyl radical scavenging assay, both HE and AD extracts showed the same capacity of scavenging, as an identical inhibition percentage was obtained, in both assay experiments, i.e., in the absence and presence of EDTA (Table 1). *T. mastichina* extracts (Table 1) produced a lower inhibition percentage of the hydroxyl radical when compared to the methanolic extract of *T. dacicus* methanolic [42], as the latter produced 50% of the radical scavenging at 18.85 μg/mL. However, the inhibition percentage of *T. mastichina* extracts was comparable to AD extracts of *T. carnosus* [9] and to HE and AD extracts of *T. pulegioides* [12].

For the scavenging of NO radical, both HE and AD extracts showed a similar efficiency (~40%, Table 1). Similar values of NO radical scavenging activity were observed for the HE extracts of *T. carnosus* [9] and for the HE and AD extracts of *T. pulegioides* [12].

The results show that, despite containing lower TPC contents (AD: 2.85 and 2.08; HE: 1.7 and 2.28 times lower than AD/HE extracts of *T. carnosus* [9] and *T. pulegioides* [12], respectively), and lower contents of individual phenolic composition (AD: 1.95; HE: 1.83 times lower than AD/HE extracts of *T. pulegioides* [12]), *T. mastichina* extracts still show a significant antioxidant activity, especially for the scavenging of hydroxyl and nitric oxide radicals. This can be due to the different individual phenolic compounds present in *T. mastichina* extracts or due to a higher synergism between the phenolic compounds present in *T. mastichina* extracts when compared to *T. carnosus* and *T. pulegioides* extracts. Overall, the results from these screening assays show that *T. mastichina* may be a potential source of phenolic compounds with relevant antioxidant activities. In addition, decoctions, the traditional method of consumption, extract a significant amount of the bioactive compounds when compared to the exhaustive HE extraction (51% of TPC was obtained with AD), and show comparable radical scavenging activities.

### 3.5. Anti-Proliferative Effect of T. mastichina Extracts

**Figure 3:**
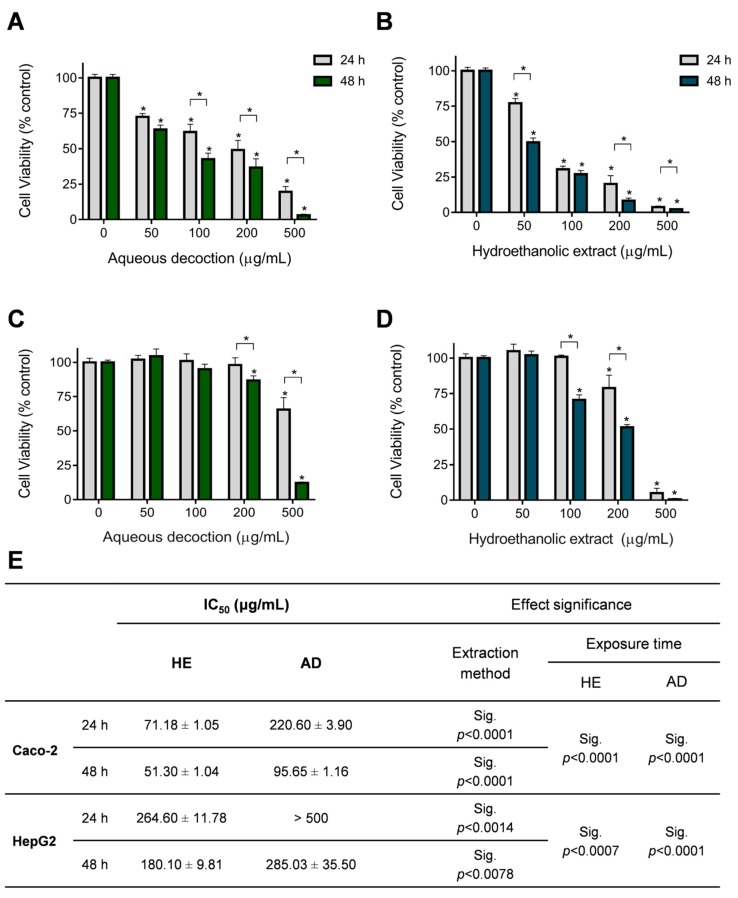
Anti-proliferative effect of *T. mastichina* extracts against Caco-2 (**A**,**B**) and HepG2 (**C**,**D**) cells. Effect of aqueous decoction (**A**,**C**) and hydroethanolic (**B**,**D**) extracts of *T. mastichina* after 24 or 48 h of exposure (as denoted). Results are expressed as (mean ± SD, *n* = 4. (*) denotes significant differences, *p* < 0.05. The calculated IC_50_ values for Caco-2 and HepG2 cells exposed to both extracts are shown in (**E**). Abbreviation: AD, aqueous decoction; HE, hydroethanolic extract; Sig., significant; n.s., not significant.

In order to evaluate the anti-proliferative effect of AD and HE extracts of *T. mastichina*, we used the Alamar Blue (AB) reduction assay and two cell lines, HepG2 and Caco-2 (please see the methods for details). Cells were exposed to different concentrations of *T. mastichina* extracts (50, 100, 200, and 500 μg/mL) for 24 or 48 h and results were compared with control cells (non-exposed cells). As shown in Figure 3, all the studied extracts had a dose- and time-dependent inhibitory effect on Caco-2 (Figure 3A,B) and HepG2 (Figure 3C,D) cell viability. HE extracts presented a higher anti-proliferative activity/cytotoxicity on Caco-2 cells than the AD extracts, which correlates with its higher (almost double) concentration of phenolic compounds (Table 2). The half-maximal inhibitory concentration (IC_50_) of HE extract was statistically significantly lower than the IC_50_ values obtained for the AD extracts of *T. mastichina* (Figure 3E), at both time points. Caco-2 cells were more sensible to *T. mastichina* extracts, compared to HepG2 cells, which is indicated by statistically significant lower IC_50_ values after 24 or 48 h of exposure (Figure 3E) for both extracts. In general, the IC_50_ values obtained after exposure of the two cell lines to *T. mastichina* extracts were lower than those reported for the extracts obtained in the same way for *T. pulegioides* [12], with the exception of Caco-2 at 24 h exposure to AD (*T. pulegioides* AD extract IC_50_ in Caco-2, at 24 h, was 137.7 ± 9.82 µg/mL [12]). In addition, the IC_50_ values obtained for the AD extracts from *T. mastichina* for both cell lines are lower than the IC_50_ values obtained for AD and HE extracts from *T. carnosus* [9]. The lower IC_50_ values obtained for the HE extracts in the HepG2 cells by Martins-Gomes et al. (2019) were attributed to the presence of significant amounts of OA and UA [6], which were not detected in the *T. mastichina* HE extracts. Although extracts of *T. carnosus* and *T. pulegioides* contain higher amounts of phenolic compounds compared to extracts from *T. mastichina*, exposure of Caco-2 and HepG2 cells to HE and AD extracts from *T. mastichina* results in significantly lower IC_50_ values compared to HE and AD extracts from *T. pulegioides* [12], and to AD extracts from *T. carnosus* [6]. Taking into account that HE extracts from *T. pulegioides* only contain 1.30 mg of salvianolic acid derivative (only salvianolic acid I being present) and that salvianolic acid derivatives are absent in the AD extract [12], the higher abundance of salvianolic acid derivatives in *T. mastichina* extracts may be responsible for the higher anti-proliferative activity of *T. mastichina* extracts. Wang et al. [43] observed that salvianolic acid B induced a time- and dose-dependent reduction in HepG2 cell proliferation. At the highest concentration tested (250 µM), the cell proliferation was reduced by 75% and 80% after 24 and 48 h of exposure, respectively [43]. In addition, salvianolic acid B was shown to inhibit the growth of several head and neck squamous carcinoma cell lines (JHU-022 and JHU-013 cells), with an IC_50_ of 18 and 50 μM, respectively [44]. Also, salvianolic acid B (125 μM) was shown to reduce the cell viability of different human cell lines, in particular, liver cell lines, Huh-7 and SK-HEP-1, to 45% and to 25% of the control [44]. Salvianolic acid A and B have been reported as good candidates against several types of cancer as these molecules target several cell mechanisms involved in apoptosis, cell cycle regulation, and inflammation [44,45,46]. As salvianolic acids act through mechanisms that modulate various signaling pathways (e.g., Mitogen Activated Protein Kinase (MAPK), phosphoinositide-3-kinase–protein kinase B/Akt (PI3K/PKB/Akt), nuclear factor kappa B (NF-κB), mammalian target of rapamycin (mTOR) pathways), which are often deregulated in cancer cells and are usually associated with drug resistance [45], plants containing these components are potential candidates as functional foods.

Nevertheless, the nature of salvianolic acid derivatives present in the extracts can also influence the anti-proliferative activity. *T. carnosus* AD extract contained 37 to 42 mg of salvianolic acid derivatives per gram of extract, with salvianolic acid A isomer (on average 53% of salvianolic acid derivatives extracted with AD) and salvianolic acid K (on average 41% of the salvianolic acid derivatives extracted with AD) as main derivatives, followed by salvianolic acid A (~6%). Extracts of *T. mastichina* contained lower amounts of salvianolic acid derivatives (27 mg/g of extract) compared to extracts from *T. carnosus*. However, the profile of individual salvianolic acid derivatives differs from the salvianolic acid derivative profile from *T. carnosus* extracts. Extracts from *T. mastichina* contained salvianolic acid A isomer as the main salvianolic acid derivative (69%), followed by salvianolic acid B/E isomer 2 (27%), and salvianolic acid K (3%), suggesting that the higher anti-proliferative activity of *T. mastichina* extracts when compared to *T. carnosus* extracts might be due to the presence of the newly tentatively identified salvianolic acid B/E isomer 2 (Table 2).

The American National Cancer Institute (Suffness and Pezzuto, 1990) and South-American Office for Anti-Cancer Drug Development (Mans et al., 2000) recommend considering crude extracts with IC_50_ values less than 30 to 50 μg/mL for further purification, and study, and use as functional foods. The HE extract from *T. mastichina* showed an IC_50_ value of about 51 µg/mL in the Caco-2 cells after 48 h (Figure 3E), and seems to be a promising source of anti-proliferative compounds against colon cancer.

## 4. Conclusions

This is the first work describing the detailed phenolic composition of exhaustive hydroethanolic and aqueous decoction extracts of *T. mastichina.* Among other common phenolic compounds normally present in *Thymus* species, salvianolic acid A isomer, only described earlier for *T. carnosus*, and another salvianolic acid isomer named B/E isomer 2, described for the first time in *T. mastichina* and *Thymus* species, were identified. Extracts of *T. mastichina* showed a significant scavenging activity of ABTS radical cation, hydroxyl, and nitric oxide radicals. Both extracts, but especially HE extract, of *T. mastichina* showed a high anti-proliferative activity against Caco-2 cells, suggesting *T. mastichina* as an interesting candidate for further investigation concerning its anti-tumoral application either as a functional food (used as decoction or tea) or as a source of bioactive ingredients. As *T. mastichina* is an endemic species to the Iberian Peninsula, as warming and drying trends are projected for the upcoming decades in Portugal [47,48] and Spain, the climate-driven modifications in *T. mastichina* phenolic composition will be explored in future studies, using plants growing in different locations and microclimates, so as to identify the best locations for its cultivation under current and future climates, i.e., the locations that will maximize the beneficial properties of its extracts.

## Figures and Tables

**Table 1:** Extraction yields, chemical composition, and antioxidant activity of extracts obtained from *Thymus mastichina*.

		Hydroethanolic Extract	Aqueous Decoction
**Extraction yield** (%, *w*/*w*)		13.78 ± 0.42	9.32 ± 1.74 *
		**Chemical composition**	
**Total phenols** (mg Caffeic acid eq./g)	**Ext.**	178.89 ± 8.89	134.76 ± 2.64 *
	**D.P.**	24.61 ± 0.67	12.51 ± 2.97 *
**Total flavonoids** (mg Catechin eq./g)	**Ext.**	184.45 ± 5.79	195.53 ± 48.78
	**D.P.**	25.44 ± 1.57	17.37 ± 1.14 *
***Ortho-*diphenols** (mg Caffeic acid eq./g)	**Ext.**	157.69 ± 19.34	107.87 ± 12.42 *
	**D.P.**	21.65 ± 2.83	10.49 ± 3.06 *
	**CAntioxidant activity**		
**ABTS•^+^** (mmol Trolox eq./g)	**Ext.**	1.48 ± 0.06	0.96 ± 0.10 *
	**D.P.**	0.20± 0.00	0.08±0.01 *
**•OH radicals + EDTA** (% inhibition)		43.22 ± 5.28	48.52 ± 4.44
**•OH radicals − EDTA** (% inhibition)		27.63 ± 2.56	28.23 ± 3.88
**NO• radicals** (% inhibition, after 120 min)		38.87 ± 4.13	38.91 ± 3.01

Abbreviations: Ext.: extract. D.P.: dry plant. For antioxidant activity, percentage of inhibition obtained for 1 mg/mL of extract. Significant statistical differences between extraction methods (*) when (*p* < 0.05).

**Table 2:** Phytochemical composition of *Thymus mastichina* aqueous decoction (AD) and hydroethanolic (HE) extracts determined by HPLC/DAD-ESI/MS.

Peak Number	Compound	R.T. (min)	ESI-MS^2^	Quantification				
				HE		AD		Extraction Method Sig.
				mg/g D.P.	mg/g Extract	mg/g D.P.	mg/g Extract	
**1**	Eriodictyol-di-*O*-hexoside	21.85 ± 0.07	[611]:449;287	n.q.	n.q.	n.q.	n.q.	
**2**	Naringenin-di-hexoside	22.12 ± 0.07	[595]:433;271	n.q.	n.q.	n.d.	n.d.	
**3**	Chlorogenic acid	23.09 ± 0.13	[353]:191;179;173;135	n.q.	n.q.	n.d.	n.d.	
**4**	Apigenin-(6,8)-*C*-diglucoside	24.63 ± 0.09	[593]:575;503;473;383 353	n.q.	n.q.	n.d.	n.d.	
**5**	Hydroxyjasmonic acid–hexoside	24.82 ± 0.06	[387]:369;225;207;163	n.q.	n.q.	n.q.	n.q.	
**6**	Caffeic acid	25.08 ± 0.10	[179]:135	n.d.	n.d.	0.21 ± 0.00	2.27 ± 0.03	*
**7**	Eriodictyol-*O*-hexoside	25.65 ± 0.04	[449]:287	n.q.	n.q.	n.q.	n.q.	
**8**	Unknown	25.67 ± 0.04	[495]:486;451;375;368	n.d.	n.d.	n.q.	n.q.	
**9**	Prolithospermic acid	28.33 ± 0.04	[357]:313;269;245;203	n.d.	n.d.	n.q.	n.q.	
**10**	Naringenin-*O*-hexoside	29.19 ± 0.17	[433]:313;271;267;137	n.d.	n.d.	n.q.	n.q.	
**11**	Quercetin-*O*-hexoside	29.66 ± 0.11	[463]:301	2.80 ± 0.15	20.34 ± 1.11	0.77 ± 0.34	8.35 ± 3.69	*
**12**	Naringenin-*O-*hexoside	29.98 ± 0.62	[433]:313;271	n.q.	n.q.	n.q.	n.q.	
**13**	Eriodictyol-*O*-hexuronide	31.95 ± 0.22	[463]:287;175	n.q.	n.q.	n.d.	n.d.	
**14**	Luteolin-*O*-hexoside	32.11 ± 0.10	[447]:285	n.q.	n.q.	n.q.	n.q.	
**15**	Salvianolic acid F derivative	32.72 ± 0.19	[375]:313;269;179;135	n.d.	n.d.	n.q.	n.q.	
**16**	Quercetin-O-hexuronide	33.29 ± 0.15	[477]:301	n.q.	n.q.	n.d.	n.d.	
**17**	Luteolin-*O*-hexoside	34.18 ± 0.18	[447]:285	2.87 ± 0.56	20.85 ± 4.08	0.87 ± 0.29	9.34 ± 3.15	*
**18**	Unknown	35.63 ± 0.20	[523]:505;477;454;391	n.q.	n.q.	n.d.	n.d.	
**19**	Salvianolic acid B/E isomer 2	36.49 ± 0.37	[717]:555;519;475;357;295	2.26 ± 0.09	16.40 ± 0.65	0.7±0.14	7.49 ± 1.55	*
**20**	Salvianolic acid A isomer	37.33 ± 0.22	[493]:383;313;295	4.20 ± 0.42	30.47 ± 3.03	1.73 ± 0.54	18.57 ± 5.74	*
**21**	Luteolin-*O*-hexorunide	37.73 ± 0.29	[461]:285;175	n.q.	n.q.	n.q.	n.q.	
**22**	Rosmarinic acid	38.65 ± 0.22	[359]:223;179;161	8.00 ± 0.92	58.06 ± 6.74	3.15 ± 1.07	33.79 ± 11.48	
**23**	Apigenin-*O*-hexoside	39.46 ± 0.23	[431]:269	n.q.	n.q.	n.q.	n.q.	
**24**	Chrysoeriol-*O*-hexoside	39.38 ± 0.16	[461]:299;160	n.q.	n.q.	n.q.	n.q.	
**25**	Salvianolic acid K	40.81 ± 0.01	[555]:537;493;359	0.57 ± 0.13	4.15 ± 0.96	0.07 ± 0.01	0.75 ± 0.07	*
**26**	Salvianolic acid I	41.24 ± 0.40	[537]:493;359	2.13 ± 0.14	15.44 ± 1.01	n.d.	n.d.	*
**27**	Quercetin-*O*-hexoside-hexuronide	42.83 ± 0.33	[639]:301	n.q.	n.q.	n.q.	n.q.	
**28**	Apigenin-*O*-hexuronide	43.59 ± 0.37	[445]:269;175	n.q.	n.q.	n.q.	n.q.	
**29**	Chrysoeriol-*O*-hexuronide	44.79 ± 0.38	[475]:299	1.05 ± 0.16	7.51 ± 1.13	0.43 ± 0.13	4.61 ± 1.35	n.s
**30**	Salvianolic acid K isomer	45.74 ± 0.64	[555]:493;359	n.q.	n.q.	n.q.	n.q.	
**31**	Luteolin	52.99 ± 0.32	[285]:241;217;199;75;151	n.q.	n.q.	n.q.	n.q.	
			**Total phenolic compounds**	23.87 ± 1.48	173.23 ± 10.74	7.93 ± 2.52	85.17 ± 27.01	*
			**Total flavonoids**	6.71 ± 0.87	48.71 ± 6.32	2.07 ± 0.76	22.30 ± 8.20	*
			**Total phenolic acids**	17.16 ± 0.61	124.52 ± 4.42	5.86 ± 1.75	62.87 ± 18.81	*

AD: aqueous decoction; HE: hydroethanolic extractions; RT: retention time; ESI-MS^2^—Fragment ions obtained after fragmentation of the pseudo-molecular ion [M]^−^; n.q.: detected but not quantified; n.d.: not detected; n.s.: not significant. Tukey’s post hoc test, significant statistical differences (*) between extraction methods, for mg/g of dry plant (D.P.), if (*p* < 0.05). Results are presented as mean ± standard deviation, *n* = 3.

## Chemical Compounds Studied in this Article

Caffeic acid (PubChem CID: 689043); Luteolin (PubChem CID: 5280445); Quercetin-3-*O*-glucoside (PubChem CID: 5280804); Rosmarinic acid (PubChem CID: 5281792); Salvianolic acid A (PubChem CID: 5281793); Salvianolic acid B (PubChem CID: 6441188).
